# Development of a valve type semi-closed extracorporeal circulation system

**DOI:** 10.1007/s10047-021-01249-5

**Published:** 2021-02-03

**Authors:** Takahiro Okumura, Keisuke Matsuda, Yu Fukuoka, Junya Dai, Naoko Shiraishi

**Affiliations:** 1 School of Clinical Engineering, Faculty of Health and Medical Care, Saitama Medical University, 1397-1, Yamane, Hidaka, Saitama 350-1241 Japan; 2 Department of Clinical Engineering, National Hospital Organization Sendai Medical Center, 2-11-12, Miyagino, Miyagino-ku, Sendai, Miyagi 983-8520 Japan; 3 Department of ME Serves, International Medical Center, Saitama Medical University, 1397-1, Yamane, Hidaka, Saitama 350-1298 Japan

**Keywords:** Cardiopulmonary bypass, Closed-type extracorporeal circulation, Safety valve, Negative pressure, Low venous return

## Abstract

In Japan, perfusionists who work on other clinical tasks are involved in cardiopulmonary bypass. Moreover, the number of cases they can perform is limited. In view of this situation, valve type semi-closed extracorporeal circulation (VACC) was developed as a system that enables extracorporeal circulation (ECC) regardless of perfusionists’ experience. The VACC circuit is based on a conventional open-type ECC circuit. A safety valve is installed at the outlet of the reservoir. It is closed by lowering the reservoir pressure below the venous circuit pressure (Pv), thereby providing a closed-type ECC in which the reservoir is separated from the venous circuit (V-circuit). A closed-type ECC needs means to cope with negative pressure generated in the V-circuit and to remove air mixed in the V-circuit. Water experiments to verify the safety of the VACC were conducted. In experiments simulating low venous return, when the Pv dropped, the safety valve opened so that the V-circuit was connected to the reservoir, and the excessive negative pressure was relieved. In the VACC circuit, a bubble trap is installed in the V-circuit, and the air is degassed to the reservoir by a roller pump (D-pump). A water experiment was conducted to verify the principle of the constant degassing method using the D-pump. It verified that the blood storage volume could be maintained constant even if the D-pump is continuously driven. The VACC system provides handling of air mixed in the V-circuit and safety in the case of low venous return.

## Introduction

Conventional cardiopulmonary bypass (CPB) in cardiac surgery is based on open-type extracorporeal circulation (ECC) using an open reservoir. In order to stabilize the patient’s hemodynamics, independent manual operations for controlling the arterial flow rate and the venous flow rate must be performed simultaneously and cooperatively. Therefore, these operations require sufficient skill.

On the other hand, in closed-type ECC, such as for extracorporeal membrane oxygenation (ECMO), the arterial flow rate and the venous flow rate are equal without advanced operating techniques. However, for use in cardiac surgery, a venous reservoir is an indispensable part in the CPB circuit to adjust the amount of circulating blood in the patient and measures must be taken to remove air mixed in the venous circuit (V-circuit). Furthermore, since closed-type ECC forcibly removes blood by a pump, there is the problem that excessive negative pressure is generated in the V-circuit when there is low venous return.

At many medical facilities in Japan, perfusionists work concurrently on other clinical tasks such as hemodialysis and medical device management. Moreover, 60% of the facilities that perform cardiac surgery using CPB in Japan have less than 100 cases per year, and 30% have less than 50 cases per year. In this situation, perfusionists strive to maintain safe CPB techniques. However, there have been several incidents related to CPB, including those where a venous reservoir was emptied in an open-type ECC circuit [1, 2].

**Fig. 1:**
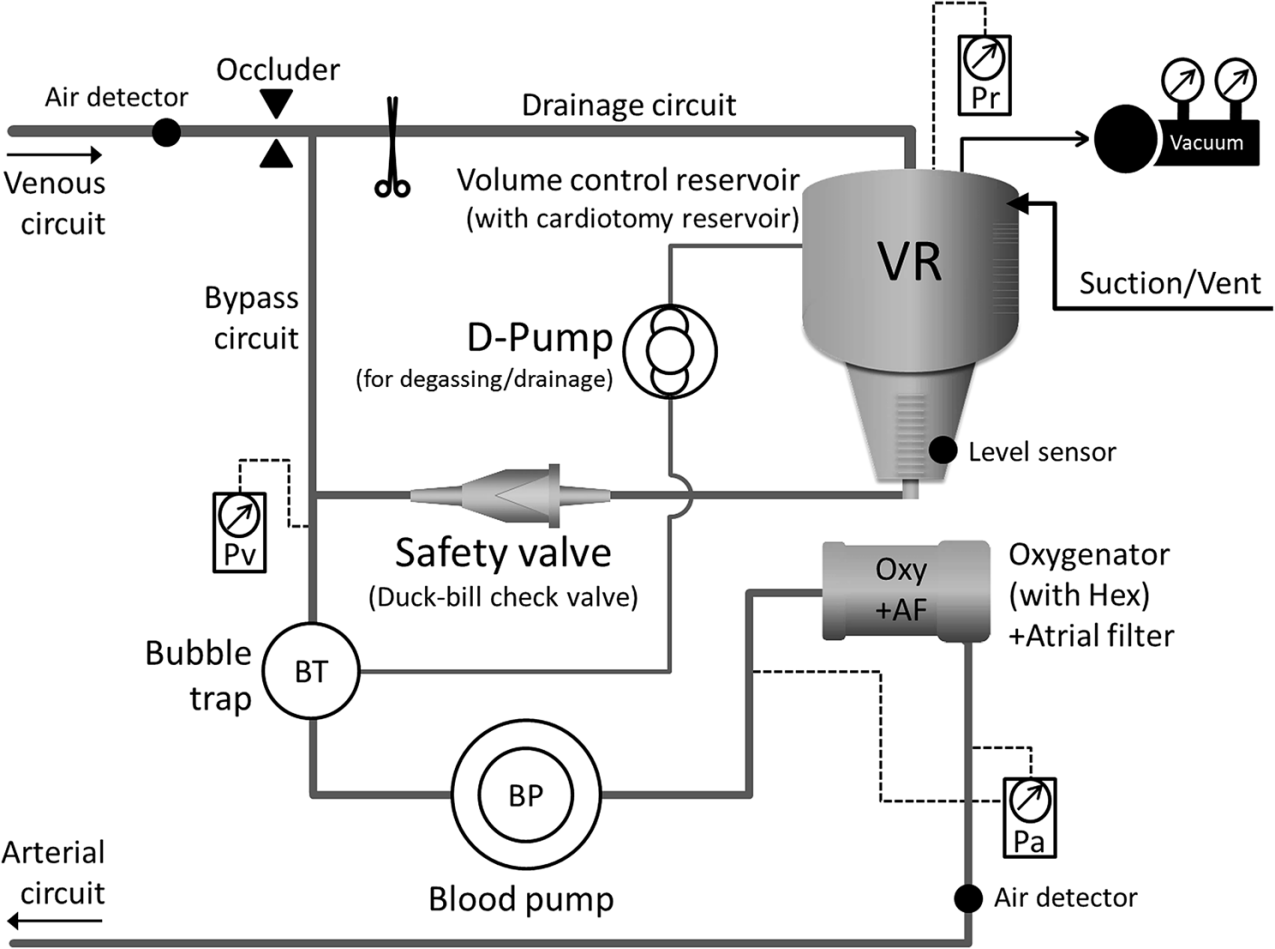
Valve type semi-closed extracorporeal circulation (VACC) system. The VACC circuit is based on a conventional open-type ECC circuit. The venous reservoir is bypassed by connecting the inlet and outlet and shutting the inlet. A safety valve is installed at the outlet of the volume control reservoir. By applying negative pressure to the reservoir, the safety valve closes. Air from the venous circuit is trapped in the bubble trap and degassed by the D-pump

Given this situation and the future, we felt the need for innovation in conventional CPB technology. Therefore, we devised a valve type semi-closed extracorporeal circulation (VACC) system and reported a prototype [3]. We have improved the circuit design so that the VACC system can be implemented with a simple circuit configuration change from a conventional open-type ECC circuit (Fig. 1). We have been developing safe CPB technology that does not depend on the perfusionist’s experience.

In this paper, the system and principle of the VACC are described, and the results of water experiments conducted to verify its safety with respect to problems with a closed-type ECC system are reported.

## Materials and methods

### VACC circuit

Figure 1 shows a schematic diagram of the VACC circuit. The VACC circuit is based on an open-type ECC circuit. The venous reservoir is bypassed by connecting the inlet and outlet and shutting the inlet. This is the volume control reservoir (VR) in Fig. 1. A safety valve is installed at the outlet of the VR. The safety valve, a duck-bill type check valve, is conventionally used to prevent retrograde flow when a centrifugal pump is used for blood return. A bubble trap is installed immediately before the systemic blood pump to trap air mixed in the V-circuit, and the air is degassed to the VR by a roller pump installed in the purge line.

### Principle of VACC

**Fig. 2:**
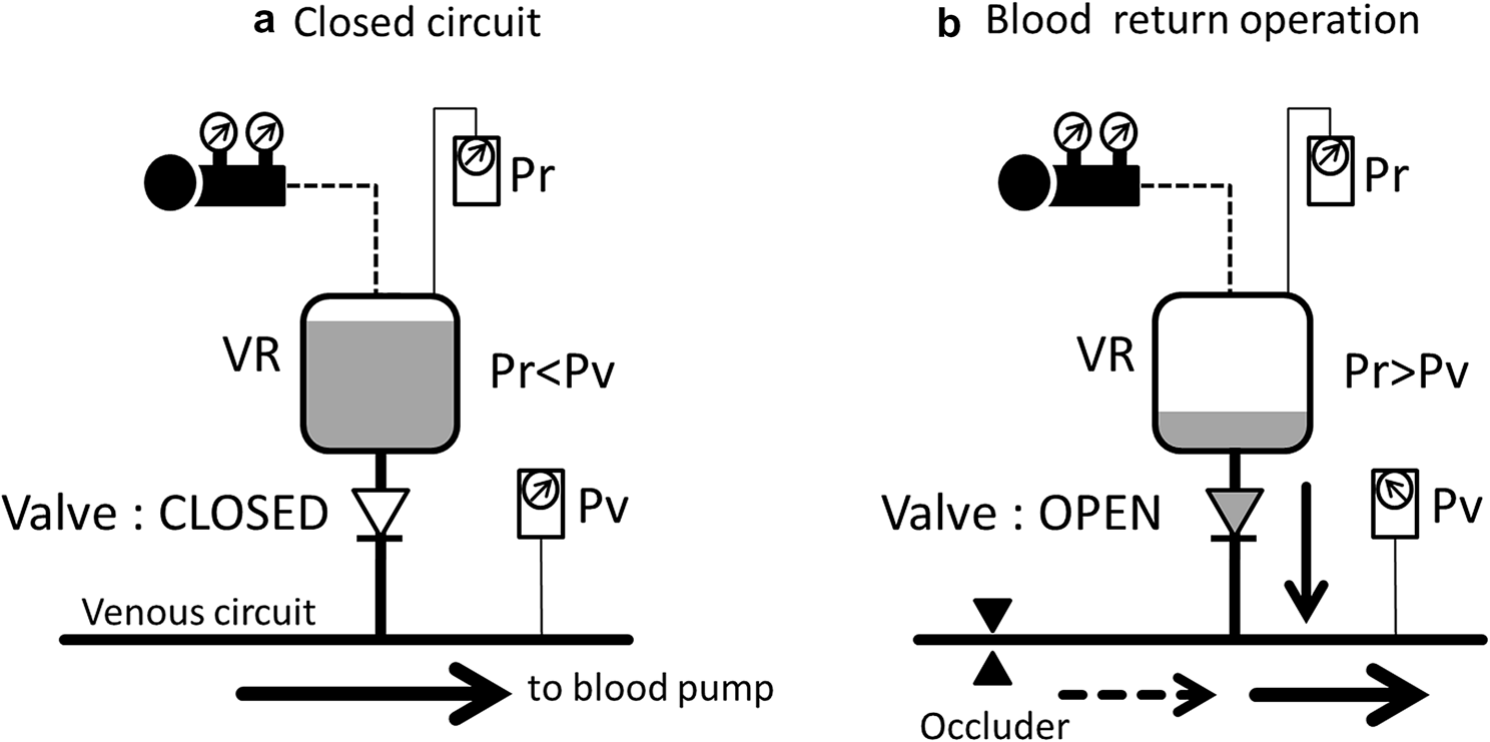
Behavior of the safety valve and how to operate the VACC. Pr is the reservoir pressure, and Pv is the venous circuit pressure. **a** The safety valve is closed when Pr is lower than Pv. **b** The safety valve is opened when the venous circuit is narrowed by an occluder or forceps. The blood in the VR returns to the circulation circuit

As shown in Fig. 2a, in principle, the safety valve at the outlet of the VR closes when the reservoir pressure (Pr) is lower than the venous circuit pressure (Pv). The VACC circuit then becomes a completely closed-type ECC similar to ECMO. For that purpose, negative pressure is supplied to the VR using a negative pressure regulator.

As shown in Fig. 2b, the Pv decreases by applying a stenosis to the V-circuit, and when the Pv drops below Pr, the safety valve opens. The blood in the VR is aspirated into the V-circuit. When the stenosis applied to the V-circuit is released, the balance between Pv and Pr returns to its original state (i.e. Pv > Pr). At this point, the safety valve closes, and the blood storage volume in the VR (BSVr) after blood return is maintained.

To drain blood into the VR to reduce the amount of circulating blood in the patient, the shut-off of the VR inlet is released with the safety valve closed. In addition, there is a method for using a pump for degassing from a bubble trap.

In a completely closed-type ECC system, excessive negative pressure generated in the V-circuit at the time of low venous return is a problem. To solve this, in the VACC circuit, the safety valve opens when the Pv decreases. Since the V-circuit is released to the VR, Pv does not become excessively negative. Furthermore, the same phenomenon as in Fig. 2b occurs when Pv decreases following the decrease in the central venous pressure (CVP). Along with this, blood in the VR is supplied to the patient without any operation by the perfusionist.

### D-Pump

The air trapped in the bubble trap is degassed to the VR by a roller pump, which is the D-pump in Fig. 1, installed in a purge line. Basically, after visually confirming that air has been trapped in the bubble trap, the D-pump is driven to perform degassing. In addition, we devised a constant degassing method using the D-pump. In this method, after the start of the CPB, the D-pump is continuously driven at a low flow rate, and air that has suddenly entered the V-circuit captured by the bubble trap is immediately removed. However, this causes a part of the circulating blood to be drained to the VR. Therefore, to maintain a constant BSVr, the blood return method shown in Fig. 2b is used. Alternatively, by reducing the negative pressure applied to the VR, the BSVr remains constant. At that time, the BSVr can be controlled by adjusting Pr.

### Verification of the effect of preventing excessive negative pressure in the V-circuit

To verify the relief effect of Pv when there is low venous return in the VACC circuit, a water experiment comparing the occurrence of excessive negative pressure in the V-circuit of the VACC and a completely closed-type ECC system was conducted. The VACC circuit was connected to a soft bag for water storage, and water was perfused at 4 L/min using a centrifugal pump. First, the immediate underside of the safety valve of the VACC circuit was shut off with forceps to form a completely closed-type ECC system. To regulate the negative pressure generated in the V-circuit, a manual stenosis was applied to the V-circuit connected to the outlet of the soft bag using an adjustable clamp. While measuring Pv, the degree of manual stenosis was increased, and the target Pv in a completely closed-type ECC system was changed to three states: − 50 mmHg, − 100 mmHg, and − 150 mmHg. Next, in each state of manual stenosis of the V-circuit, the blockage just below the safety valve was released and switched to the VACC. Since the safety valve opens when Pv is lower than Pr, the water in the VR is aspirated into the circulation circuit. Therefore, the water in the soft bag was pumped to the VR using another roller pump, and the BSVr was maintained constant. The Pv at that time was recorded as the Pv in the VACC circuit. The experiment was performed by changing the Pr to − 60 mmHg, − 40 mmHg, and − 20 mmHg.

### Experiment verifying the constant degassing method using the D-pump

A water experiment was conducted to verify the principle of the constant degassing method using the D-pump. The VACC circuit was connected to a soft bag for water storage. A centrifugal pump was used for perfusion of the experimental circuit, and the rotation speed was maintained at 1500 rpm. The suction flow rate by the D-pump was 500 mL/min. The BSVr when starting the drive of the D-pump was standardized to 1000 mL. The BSVr maintained under the following experimental conditions was measured. The measurement was conducted three times, and its reproducibility was verified. As an experimental condition on the upstream side of the safety valve, the Pr was changed to − 30 mmHg, − 20 mmHg, and − 10 mmHg. As a condition on the downstream side of the safety valve, the height of the soft bag for water storage was adjusted to change the head pressure at the safety valve outlet. The head pressure measured with the circulation of the experimental circuit stopped was changed to 30 mmHg, 25 mmHg, and 20 mmHg.

## Results

### Relieving excessive negative pressure in the V-circuit

**Fig. 3:**
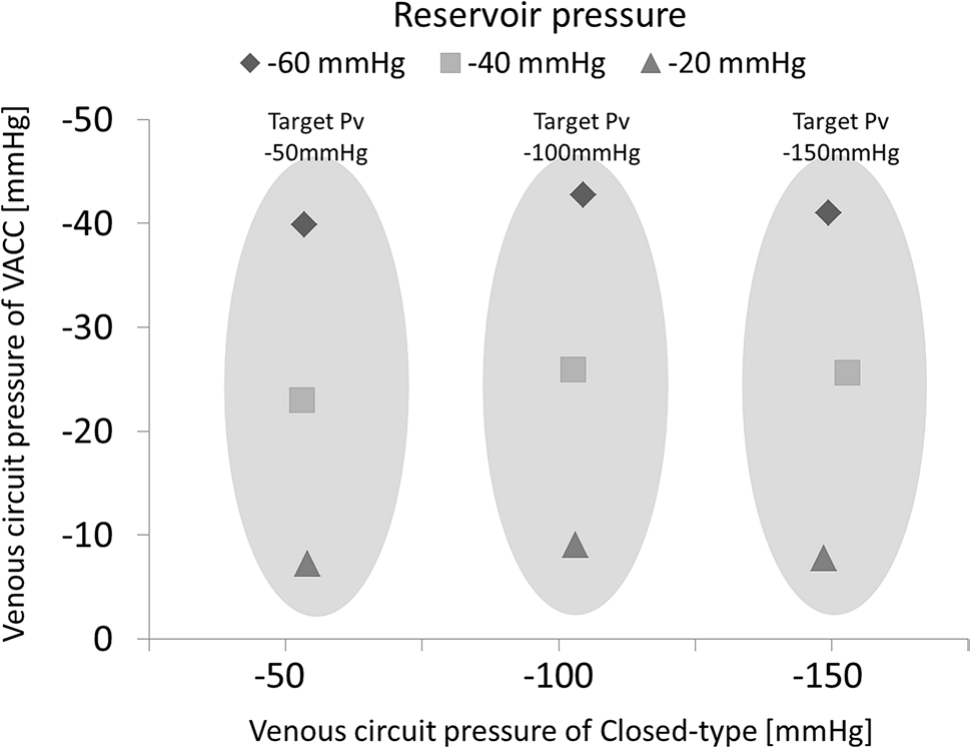
Comparison of the venous circuit pressure (Pv) with the VACC and a completely closed-type ECC during simulation of low venous return. As the stenosis of the venous circuit increases, the Pv in the completely closed-type ECC decreases. In the VACC, the excessive negative pressure is relieved, and the Pv is limited to the reservoir pressure

Figure 3 shows a comparison of the Pv of the VACC circuit and of the completely closed-type ECC system when simulating low venous return. The horizontal axis shows the Pv of the completely closed-type ECC (Pclose) in three states of manual stenosis of the V-circuit to adjust to the target Pv, and the vertical axis shows the Pv of the VACC circuit (Pvacc) for each negative pressure applied to the VR. At the target Pv of − 50 mmHg, for Pr values of − 20 mmHg, − 40 mmHg, and − 60 mmHg, Pclose/Pvacc was − 53.9 mmHg/− 7.2 mmHg, − 53.1 mmHg/− 22.9 mmHg, and − 53.4 mmHg/− 40.0 mmHg, respectively. For the same Pr values, at the target Pv of − 100 mmHg, the Pv values were − 103.1 mmHg/− 9.1 mmHg, − 102.7 mmHg/− 25.9 mmHg, and − 104.6 mmHg/− 42.8 mmHg. Similarly, at the target Pv of − 150 mmHg, the Pv values were − 148.7 mmHg/− 7.8 mmHg, − 153.1 mmHg/− 25.6 mmHg, and − 149.5 mmHg/− 41.0 mmHg, respectively. For each of the target Pv values, Pvacc was significantly higher than Pclose. In addition, the mean value ± standard deviation of Pvacc for the three target Pv values was − 8.0 ± 0.97 mmHg when the Pr was − 20 mmHg, − 24.8 ± 1.65 mmHg when the Pr was − 40 mmHg, and − 41.3 ± 1.42 mmHg when the Pr was − 60 mmHg. The results of the Pvacc for each Pr were almost the same. On the other hand, the reason why the Pvacc and the Pr were not exactly the same is considered to be due to the effect of the head pressure upstream of the safety valve. According to this experiment, as the manual stenosis of the V-circuit increased, Pclose decreased, whereas in the VACC circuit, the excessive negative pressure of the V-circuit was relieved, and Pvacc was limited to Pr.

### The constant degassing method using the D-pump

**Fig. 4:**
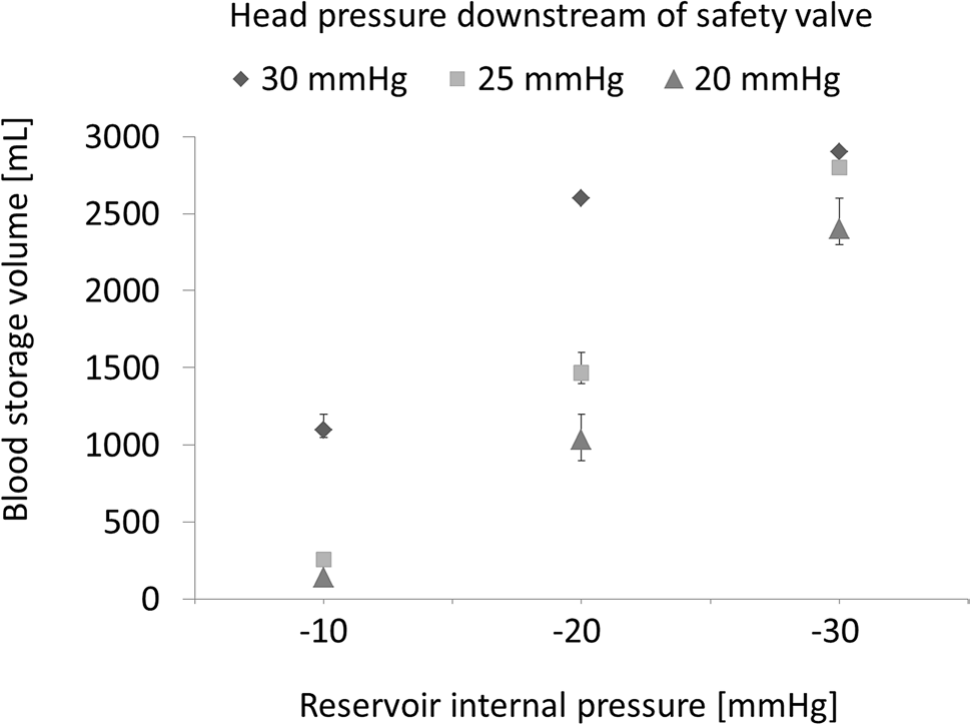
Constant degassing method using the D-pump. The arterial and venous circuits of the VACC are connected to a soft bag. The centrifugal pump is perfused at a rotation speed of 1500 rpm. Even when the D-pump continues to aspirate at 500 mL/min from the bubble trap, the blood storage volume is maintained constant according to the reservoir pressure

When it was aspirated from the bubble trap with the D-pump, the BSVr increased or decreased gradually, and it was visually confirmed that the BSVr was maintained constant after about 5 min at the longest. Figure 4 shows the mean value and maximum/minimum values of three results of the BSVr measurements. When the head pressure applied downstream of the safety valve was 30 mmHg, the mean value ± standard deviation (minimum–maximum) was 2900 ± 0 mL when the Pr was − 30 mmHg, 2600 ± 0 mL when the Pr was − 20 mmHg, and 1100 ± 70.7 mL (1050–1200 mL) when the Pr was − 10 mmHg. The average perfusion rate was 5.06 L/min. Similarly, when the head pressure was 25 mmHg, it was 2800 ± 0 mL when the Pr was − 30 mmHg, 1467 ± 94.3 mL (1400–1600 mL) when the Pr was − 20 mmHg, and 257 ± 4.7 mL (250–260 mL) when the Pr was − 10 mmHg. The average perfusion rate was 5.15 L/min. When the head pressure was 20 mmHg, it was 2400 ± 141.4 mL (2300−2600 mL) when the Pr was − 30 mmHg, 1033 ± 124.7 mL (900–1200 mL) when the Pr was − 20 mmHg and 140 ± 0 mL when the Pr was − 10 mmHg. The average perfusion rate was 5.15 L/min.

## Discussion

The features of the VACC circuit are: (1) a closed-type ECC circuit that can adjust the circulating blood volume in a patient; (2) the D-pump can remove air in the V-circuit; and (3) the safety valve does not generate excessive negative pressure in the V-circuit.

Since the VACC is a closed-type ECC circuit, it does not require operations to maintain the balance between the arterial flow rate and the venous flow rate. Weaning from CPB is a time period that is sensitive to the patient’s hemodynamic changes, and its stability is absolutely necessary. For that purpose, it is important to monitor and understand the patient’s hemodynamics. Compared to conventional open-type ECC, the operation of weaning with the VACC is easy, so the perfusionist can concentrate on hemodynamic management.

In comparison with an open-type ECC, what is needed in a closed-type ECC is a means to remove air mixed in the V-circuit. Recently, the uses of closed-type minimal extracorporeal circuits (MECCs) are expanding in cardiac surgery. Roosenhoff et al. [4] reported that incorporating a venous bubble trap (VBT) into the MECC system reduced gaseous microemboli in CPB compared to standard MECCs without a VBT. Similarly, the VACC circuit uses a bubble trap to handle air that has entered the V-circuit. Moreover, the air trapped in the bubble trap is degassed to the VR by the D-pump. Therefore, the VACC system is safe against air mixed in the V-circuit. Depending on the CPB system, there is a function for interlocking the driving of an air detector and a roller pump, so the air can be automatically degassed. Furthermore, we devised a constant degassing method using the D-pump. In this method, when the D-pump is driven, the circulating blood of the patient moves to the VR. However, this works to lower the Pv, so the safety valve opens, and blood in the VR is returned to the V-circuit. If the flow rate of the D-pump and the flow rate return from the VR are balanced, the BSVr will be kept constant. If it is assumed that CPB must be continued under continuous air mixing more than the amount removed by the D-pump suction, the VACC circuit can be switched to an open-type ECC by replacing the forceps at the inlet of the VR with the bypass circuit.

A closed-type ECC has the disadvantage that it can create excessive negative pressure in the V-circuit. Momose et al. [5], who are using a closed-type ECC circuit clinically, have reported excessive negative pressure in the V-circuit during CPB for coronary artery bypass graft surgery. As a countermeasure, when Pv decreases, the arterial flow rate is temporarily reduced. Furthermore, when CVP decreases, the dedicated pump for controlling blood storage volume is operated to increase the patient’s circulating blood volume. Compared to that, the VACC is safer because of the safety valve. In other words, the VACC has the advantage in that there is no excessive negative pressure inside the V-circuit. Moreover, when CVP decreases, Pv begins to decrease, the safety valve opens, and the blood in the VR is supplied to the patient without any manual operation.

The Pv at which the safety valve of the VACC opens depends on the Pr. If the Pr is set to be low, the safety valve becomes difficult to open, and the feature of a closed-type ECC is stabilized, but the effect of relieving the Pv is weakened. If the Pr is set to be high, the safety valve is easily opened, the excessive negative pressure of the V-circuit is less likely to occur, and responsiveness to supplying blood to the patient is good. However, the characteristic as a closed-type ECC is diminished. We assume that the VR should be installed at a height where it can be easily seen by the perfusionist, and the Pr should be set to a negative pressure that functions stably as a closed-type ECC.

As described above, the BSVr may decrease due to the Pv. Therefore, it should be noted that the VACC may draw air from the VR. To prevent this, a level sensor should be installed on the VR.

**Fig. 5:**
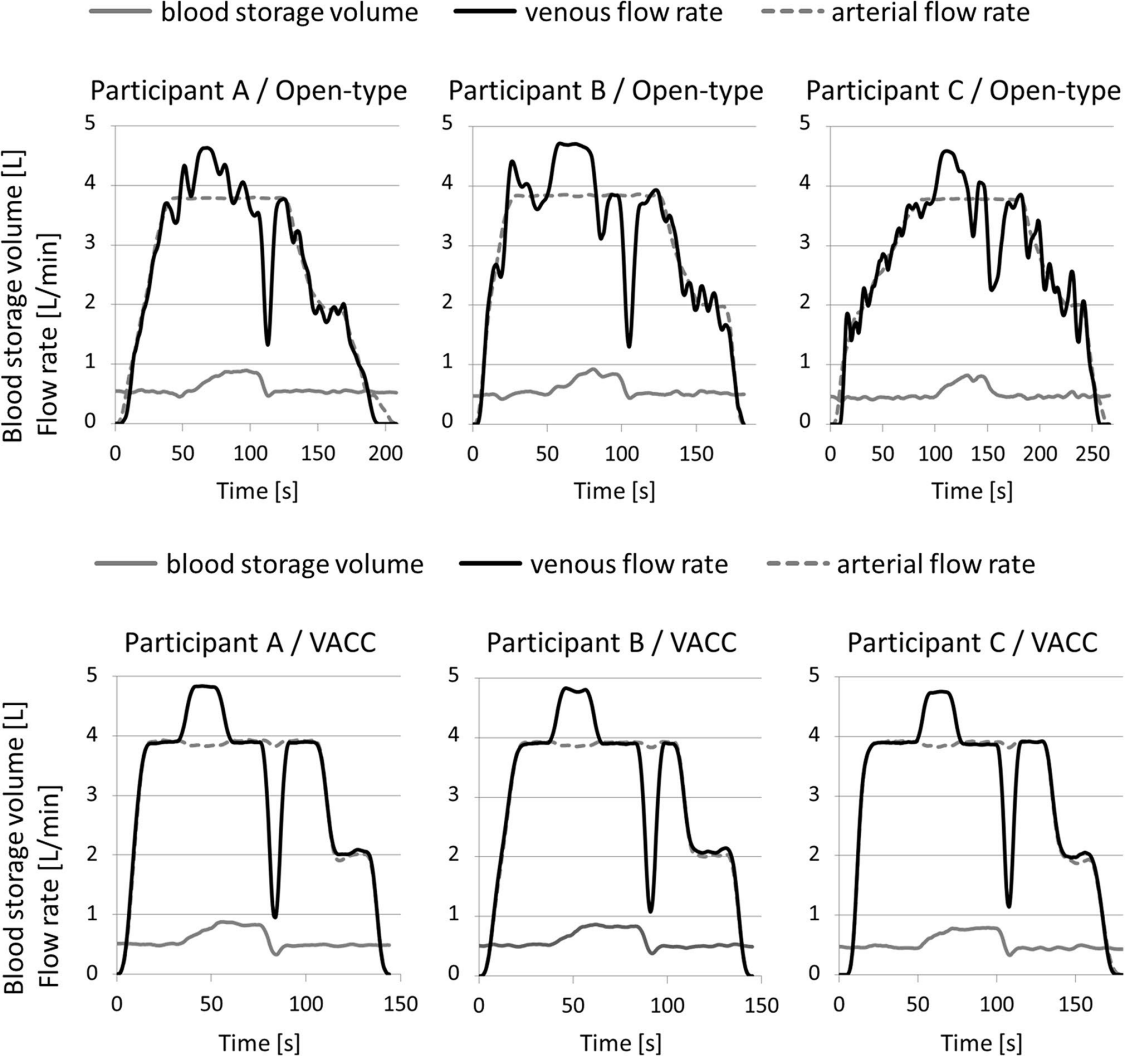
Time courses of blood storage volume and flow rate in the open-type ECC and the VACC system in a demonstration. Participant A is a perfusionist with conventional CPB experience. Participant B has no CPB experience, but has experience practicing operations with mock circuits of both an open-type and the VACC. Participant C has no CPB experience and no experience in operating simulations of both an open-type and the VACC. These results were presented at the 2nd meeting of the Federation of Asian Perfusion Societies in 2015

Compared to conventional open-type ECC, the advantage of the VACC is experience-independent operability of CPB. We held demonstrations of the VACC with mock circuits for participants with different levels of CPB experience and presented the results at the 2nd meeting of the Federation of Asian Perfusion Societies in 2015. Figure 5 shows the results that were recorded by an ECC simulator with an open-type ECC and with the VACC. Participants performed the same simple scenario. The operation of the VACC resulted in little difference between participants. Due to the experience-independent operability of the VACC, it may be possible to shorten the training time for a perfusionist. This is because the trainee can directly perform the instructor’s directions during their operation. In Japan, a clinical engineer (CE) with a national qualification operates the CPB system. Most CE training schools do not have a sufficient environment and time for hands-on training in CPB, including clinical training, because trainees have to learn the technologies of other medical devices. Therefore, training as a perfusionist begins after starting employment at a medical facility. Such training is mainly on-the-job training, but the number of cases requiring CPB is small at many facilities in Japan. In addition, when a trainee operates the CPB, a veteran CE is required as the instructor, and the number of other personnel available for clinical work is reduced. This inefficient utilization of human resources continues until the trainee becomes independent. In view of this situation, Horiguchi et al. [6] devised a new operation method for open-type ECC to install a flow meter on the V-circuit. The motive for introducing the new method was the same as ours, and it can be seen that many facilities have the same problems. Although the VACC requires some circuit changes from the conventional circuit, we expect that the introduction of the VACC will be an effective use of limited human resources and will make it possible to use human resources while containing labor costs.

## Conclusion

Considering the current situation and future of the medical field in Japan, we feel the need for innovation in conventional CPB. Therefore, we have developed the VACC with operability that does not depend on the perfusionist’s experience level. The VACC is a closed-type ECC that can be used in cardiac surgery. For this purpose, it provides adjustment of circulating blood volume, handling of air mixed in the V-circuit, and protection against excessive negative pressure in the V-circuit when there is low venous return. The VACC is expected to become the next-generation CPB system, which helps in the unique situation of Japan where the opportunities for perfusionists to gain experience are limited.
